# Evaluation of Positive End-Expiratory Pressure Strategies in Patients With Coronavirus Disease 2019–Induced Acute Respiratory Distress Syndrome

**DOI:** 10.3389/fmed.2021.637747

**Published:** 2021-07-20

**Authors:** Chun Pan, Cong Lu, Xiaobin She, Haibo Ren, Huazhang Wei, Liang Xu, Yingzi Huang, Jia'an Xia, Yuetian Yu, Lu Chen, Bin Du, Haibo Qiu

**Affiliations:** ^1^Jiangsu Provincial Key Laboratory of Critical Care Medicine, Department of Critical Care Medicine, School of Medicine, Zhongda Hospital, Southeast University, Nanjing, China; ^2^Department of Critical Care, Keenan Research Centre and Li Ka Shing Knowledge Institute, St Michael's Hospital, Toronto, ON, Canada; ^3^Interdepartmental Division of Critical Care Medicine, University of Toronto, Toronto, ON, Canada; ^4^Department of Critical Care Medicine, Qinghai University Affiliated Hospital, Xining, China; ^5^Department of Critical Care Medicine, Wuhan Asia General Hospital, Wuhan, China; ^6^Department of Critical Care Medicine, Jinggangshan University Affiliated Hospital, Jinggangshan, China; ^7^Department of Critical Care Medicine, Wuhan Wuchang Hospital, Wuhan, China; ^8^Department of Critical Care Medicine, Wuhan Jinyintan Hospital, Wuhan, China; ^9^Department of Critical Care Medicine, School of Medicine, Ren Ji Hospital, Shanghai Jiao Tong University, Shanghai, China; ^10^Medical Intensive Care Medicine, Peking Union Medical College Hospital, Peking Union Medical College, Chinese Academy of Medical Sciences, Beijing, China

**Keywords:** acute respiratory distress syndrome, coronavirus disease 2019, positive end-expiratory pressure, mechanical ventilation, lung injury

## Abstract

**Background:** Different positive end-expiratory pressure (PEEP) strategies are available for subjects with coronavirus disease 2019 (COVID-19)–induced acute respiratory distress syndrome (ARDS) requiring invasive mechanical ventilation. We aimed to evaluate three conventional PEEP strategies on their effects on respiratory mechanics, gas exchanges, and hemodynamics.

**Methods:** This is a prospective, physiologic, multicenter study conducted in China. We recruited 20 intubated subjects with ARDS and confirmed COVID-19. We first set PEEP by the ARDSnet low PEEP–fraction of inspired oxygen (FIO_2_) table. After a recruitment maneuver, PEEP was set at 15, 10, and 5 cm H_2_O for 10 min, respectively. Among these three PEEP levels, best-compliance PEEP was the one providing the highest respiratory system compliance; best-oxygenation PEEP was the one providing the highest PaO_2_ (partial pressure of arterial oxygen)/FIO_2_.

**Results:** At each PEEP level, we assessed respiratory mechanics, arterial blood gas, and hemodynamics. Among three PEEP levels, plateau pressure, driving pressure, mechanical power, and blood pressure improved with lower PEEP. The ARDSnet low PEEP–FIO_2_ table and the best-oxygenation strategies provided higher PEEP than the best-compliance strategy (11 ± 6 cm H_2_O vs. 11 ± 3 cm H_2_O vs. 6 ± 2 cm H_2_O, *p* = 0.001), leading to higher plateau pressure, driving pressure, and mechanical power. The three PEEP strategies were not significantly different in gas exchange. The subgroup analysis showed that three PEEP strategies generated different effects in subjects with moderate or severe ARDS (*n* = 12) but not in subjects with mild ARDS (*n* = 8).

**Conclusions:** In our cohort with COVID-19–induced ARDS, the ARDSnet low PEEP/FIO_2_ table and the best-oxygenation strategies led to higher PEEP and potentially higher risk of ventilator-induced lung injury than the best-compliance strategy.

**Clinical Trial Registration:** www.ClinicalTrials.gov, identifier: NCT04359251.

## Introduction

The World Health Organization announced the coronavirus disease 2019 (COVID-19) outbreak a pandemic on March 11, 2020. It has been reported that 67% of critically ill subjects with COVID-19 developed acute respiratory distress syndrome (ARDS) requiring invasive mechanical ventilation (1). Setting a sufficient positive end-expiratory pressure (PEEP) level is crucial for improving oxygenation, keeping alveoli open, and reducing cyclic reopening–closing (“atelectrauma”) (2, 3). However, an unnecessarily high PEEP can increase the risk of overdistension, especially in subjects with low recruitability and severe lung inhomogeneity (4–8).

Determining the appropriate or “best” PEEP is, however, challenging (9). Several methods have been used in clinical trials and routine practice. ARDSnet low and high PEEP–fraction of inspired oxygen (Fio_2_) tables are probably the most widely used methods in randomized controlled trials (10, 11). These tables, aiming at maintaining oxygenation while minimizing the use of high Fio_2_, provided great feasibility for clinical practice. Alternatively, clinicians can perform a decremental PEEP trial (12), to find the PEEP providing the highest compliance (“best-compliance” strategy) or providing the highest ratio of partial pressure of arterial oxygen (Pao_2_) to Fio_2_ (“best-oxygenation” strategy) (13–15). The so-called best-oxygenation strategy has not been used in large clinical trials but might be widely embedded in clinical practice where the oxygen saturation by pulse oximetry is often used as a surrogate.

While the current guidelines suggest using higher PEEP (>10 cm H_2_O) in subjects with COVID-19–induced ARDS (16), varied lung recruitability and responses to PEEP were reported from monocenter studies (17–19). Meanwhile, Marini and Gattinoni proposed that COVID-19–induced ARDS is probably different from conventional ARDS (20–22). It is unclear how those conventional PEEP strategies perform in this particular population. We thus want to evaluate three conventional PEEP strategies (ARDS low PEEP–Fio_2_ table, best-compliance, and best-oxygenation strategies) in subjects with COVID-19–induced ARDS, to see whether they result in different PEEP settings, respiratory mechanics, gas exchange, hemodynamics, and potential risk of ventilator-induced lung injury (VILI).

## Materials and Methods

### Study Population

This is a prospective physiologic study conducted in seven intensive care units (ICUs) of seven hospitals (Wuhan Jinyintan Hospital, The People's Hospital of Wuhan Xinzhou, Huangshi Hospital of Traditional Chinese Medicine, Wuhan Asia General Hospital, Wuhan Fifth Hospital, Wuhan Wuchang Hospital, and Wuhan Pulmonary Hospital, Wuhan, China) from March 5 to 16, 2020. Inclusion criteria were between 18 and 80 years old, laboratory-confirmed COVID-19, receiving invasive mechanical ventilation, and meeting the Berlin Definition of ARDS at clinical PEEP level. Exclusion criteria were pregnancy, hemodynamic instability (i.e., norepinephrine >0.05 μg/kg per minute or dopamine >5 μg/kg per minute), acute brain injury, pneumothorax, or pneumomediastinum.

The study was approved by the local Research Ethics Board. Written informed consent was obtained from substitute decision makers. The study was registered at ClinicalTrials.gov (NCT04359251).

### Study Protocol

During the study, subjects were measured at a semirecumbent position under volume control ventilation with a square flow waveform. Tidal volume (*V*_T_) was kept at 6 ml/kg per predicted body weight, and respiratory rate was kept the same at different PEEP levels. Subjects were administered a continuous infusion of analgesia and sedation. If spontaneous breathing effort was strong during sedation, neuromuscular-blocking agents were administered to suppress spontaneous breathing.

**Figure 1:**
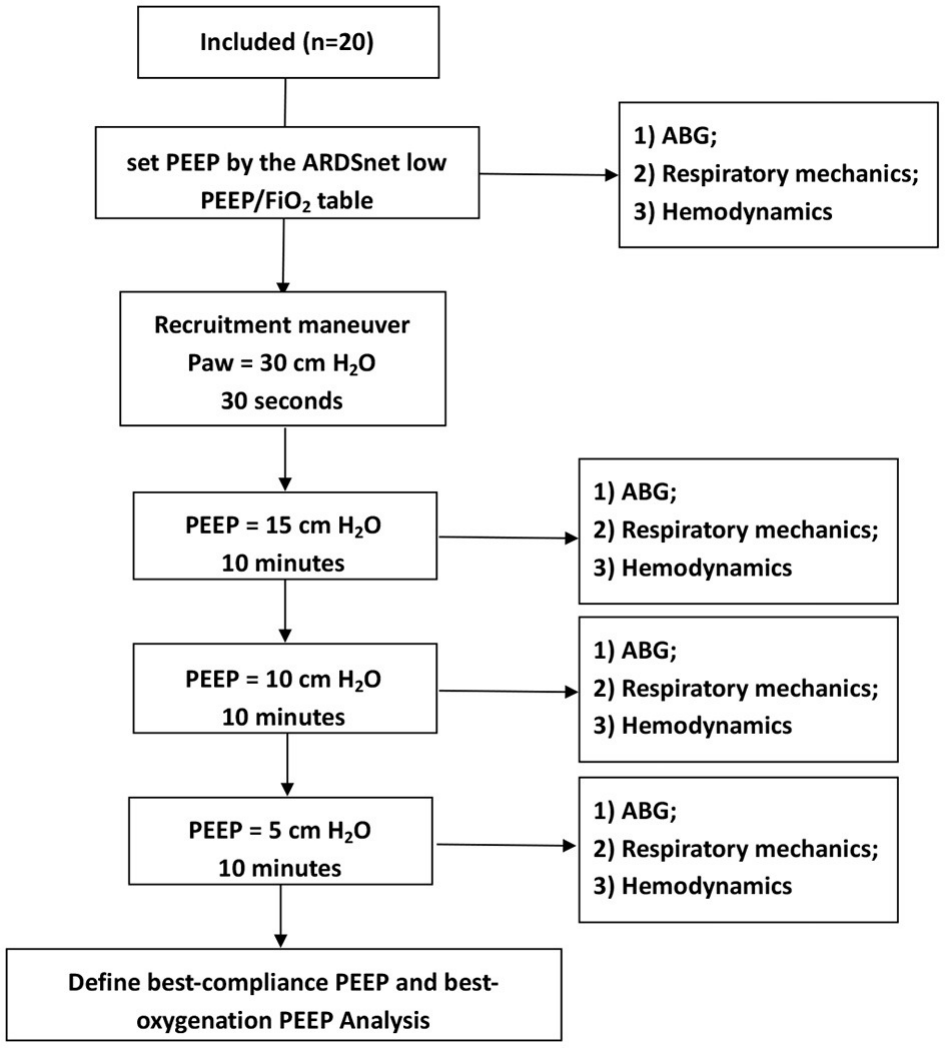
Flow diagram.

PEEP trial: (1) We first set PEEP by the ARDSnet low PEEP/Fio_2_ table; goals of PEEP and Fio_2_ settings were Pao_2_ 55-80 mm Hg, or SpO_2_ 88-95%. An arterial blood gas (ABG) was obtained at this PEEP level. (2) We then performed a recruitment maneuver by using continuous positive airway pressure at 30 cm H_2_O for 30 s, to standardize lung volume history. (3) Thereafter, PEEP was set at 15, 10, and 5 cm H_2_O for 10 min, respectively. At each PEEP level, an ABG was obtained. In addition, if a subject had extremely high plateau pressure (Pplat, e.g., >35 cm H_2_O) or hemodynamic instable or refractory desaturation, the duration of that PEEP level was reduced for safety consideration (Figure 1).

“Best-compliance PEEP” was defined as the PEEP (among 15, 10, and 5 cm H_2_O) providing the highest respiratory system compliance (*C*_rs_).

“Best-oxygenation PEEP” was defined as the PEEP (among 15, 10, and 5 cm H_2_O) providing the highest Pao_2_/Fio_2_ ratio.

### Measurements

Airway pressure was measured using a ventilator (SV300, Mindray, China). Pplat was measured by performing end-inspiratory occlusion. Total PEEP (PEEP_tot_) was measured by performing end-expiratory occlusion. Static *C*_rs_ was calculated with the following formula: *V*_T_/(Pplat – PEEP_tot_) (23). Driving pressure was calculated with the following formula: Pplat – PEEP_tot._ Mechanical power was calculated with the following formula: 0.098 × RR × *V*_T_ × (Ppeak – × driving pressure) (24).

### Criteria of “High Risk of VILI”

To determine how many subjects were potentially exposed to high risk of VILI by three PEEP strategies, we defined the high risk of VILI by meeting one of the following criteria: (1) Pplat > 30 cm H_2_O, (2) driving pressure > 15 cm H_2_O, and (3) mechanical power > 25 J/min (11, 25, 26).

### Data Collection

The general characteristics of subjects such as gender, age, height, Sequential Organ Failure Assessment (SOFA) score, and days of invasive mechanical ventilation were collected. Ventilator settings, mechanics parameters, ABG, blood pressure, and heart rate were also documented at study enrollment and during the PEEP trial.

### Statistical Analysis

Data are presented as the mean ± standard deviation, unless specified otherwise. Comparisons between three PEEP levels were conducted by using analysis of variance with repeated measures or paired *t*-test, when appropriate. Comparisons between two groups (separated by Pao_2_/Fio_2_) were conducted by using independent *t*-test. *p* < 0.05 was considered statistically significant. SPSS 20.0 (Statistical Product and Service Solutions, Chicago, IL, USA) was used for statistical analysis.

## Results

Twenty subjects (12 men and eight women, aged 64 ± 7 years, SOFA score 11 ± 2) were enrolled. The subjects received various durations of invasive mechanical ventilation before measurement (11 ± 6 days). ICU mortality was 60% (12/20). The detailed characteristics including the baseline respiratory mechanics at clinical PEEP level are reported in Table 1.

**Table 1:** Characteristics of COVID-19 patients with mechanical ventilation.

**Characteristics**	**Overall (*n* = 20)**	**Mild ARDS (*n* = 8)**	**Moderate/severe ARDS (*n* = 12)**	P^*^
Males—*n* (%)	12 (60)	6 (75)	6 (50)	0.26
Age (years)	64 ± 7	63 ± 9	65 ± 6	0.38
Height (cm)	170 ± 9	174 ± 8	167 ± 8	0.07
SOFA	11 ± 2	11 ± 1	11 ± 3	0.59
IMV (days)	11 ± 6	10 ± 8	11 ± 4	0.77
V_T_ (ml/kg)	5.6 ± 0.8	5.6 ± 0.7	5.6 ± 0.9	0.88
FiO_2_	0.66 ± 0.30	0.53 ± 0.22	0.74 ± 0.30	0.09
pH	7.36 ± 0.08	7.37 ± 0.05	7.35 ± 0.10	0.54
PaO_2_(mm Hg)	108 ± 53	138 ± 57	88 ± 41	0.03
PaO_2_/FiO_2_(mm Hg)	180 ± 75	259 ± 25	126 ± 39	<0.001
PaCO_2_(mm Hg)	60 ± 18	62 ± 20	58 ± 17	0.67
P_plat_(cm H_2_O)	23 ± 6	22 ± 7	24 ± 5	0.50
PEEP (cm H_2_O)	6 ± 2	6 ± 2	6 ± 2	0.97
ΔP (cm H_2_O)	17 ± 5	16 ± 7	17 ± 4	0.50
Crs(ml/cm H_2_O)	23 ± 8	27 ± 9	20 ± 6	0.05
MAP (mm Hg)	84 ± 12	88 ± 13	81 ± 10	0.22
HR (bpm)	95 ± 18	93 ± 15	97 ± 20	0.62

*COVID-19, Coronavirus disease 2019; SOFA, sequential organ failure assessment; IMV, invasive mechanical ventilation; V_T_, tidal volume; Pplat, plateau pressure; PEEP, positive end-expiratory pressure; ΔP, driving pressure; Crs, respiratory system compliance; MAP, mean arterial pressure; HR, heart rate*.

* *Patients with mild ARDS vs. patients with moderate or severe ARDS. Compared by t-test*.

### Responses to 15, 10, and 5 cm H_2_O of PEEP

**Figure 2:**
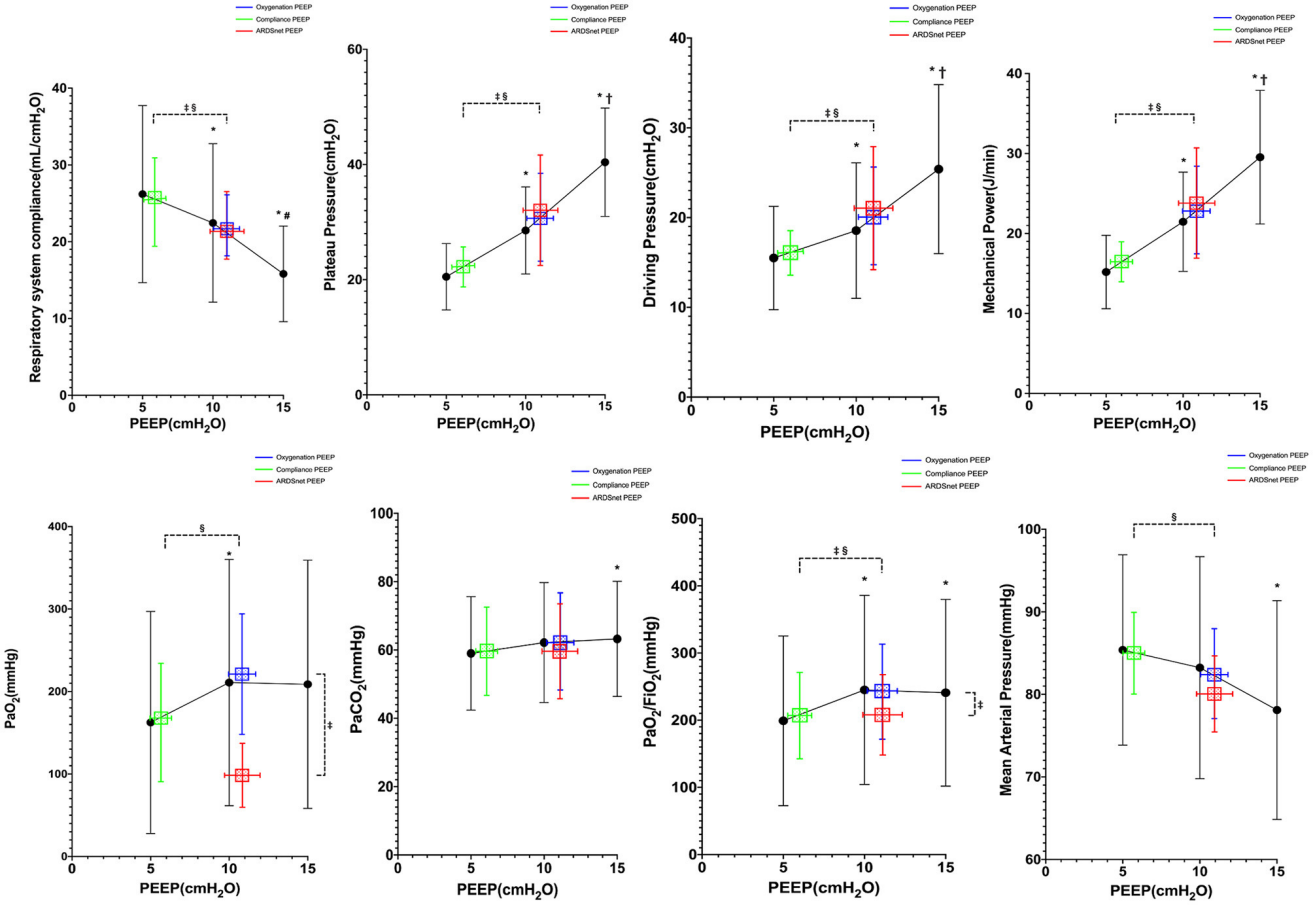
Reponses to PEEP in all subjects (*n* = 20). *vs. PEEP 5 cm H_2_O, *p* < 0.05; ^†^vs. PEEP 10 cm H_2_O, *p* < 0.05; ^‡^vs. oxygenation PEEP, *p* < 0.05; ^§^vs. ARDSnet PEEP, *p* < 0.05.

As shown in Figure 2, Pplat, driving pressure, and mechanical power were significantly decreased when PEEP was reduced from 15 to 10 cm H_2_O and from 10 to 5 cm H_2_O. We failed to obtain ABG at PEEP 15 cm H_2_O in three subjects because of extremely high Pplat (>50 cm H_2_O) at high PEEP. After the recruitment maneuver, Pao_2_/Fio_2_ was similar at 15 and 10 cm H_2_O of PEEP but dropped at 5 cm H_2_O of PEEP (*p* = 0.005). Paco_2_ was significantly higher at 15 cm H_2_O of PEEP than at 10 and 5 cm H_2_O of PEEP (*p* = 0.018). Heart rate was similar at three PEEP levels, but mean arterial pressure (MAP) was lower at 15 cm H_2_O of PEEP (*p* = 0.012).

### Comparisons of Three PEEP Strategies

As shown in Figure 2, the PEEP guided by best compliance was lower than the PEEP guided by best oxygenation and the PEEP guided by ARDSnet low PEEP/Fio_2_ table (6 ± 2 vs. 11 ± 4 and 11 ± 6 cm H_2_O, *p* = 0.001). Pplat, driving pressure, and mechanical power by best-compliance strategy were lower than those by best-oxygenation strategy and ARDSnet low PEEP–Fio_2_ table (Pplat: 21 ± 6 vs. 31 ± 11 and 32 ± 15 cm H_2_O, *p* <0.001; driving pressure: 15 ± 6 vs. 20 ± 9 and 21 ± 10 cm H_2_O, *p* < 0.001; mechanical power: 15.9 ± 4.5 vs. 23.5 ± 9.6 and 22.9 ± 10.9 J/min, *p* = 0.001). *C*_rs_ by best-compliance strategy were higher than those by best-oxygenation strategy and ARDSnet low PEEP–Fio_2_ table (26.8 ± 11.8 vs. 20.6 ± 8.0 and 20.3 ± 9.3 mL/cm H_2_O, *p* <0.01). Pao_2_ by ARDSnet low PEEP–Fio_2_ table was lower than those by best-compliance strategy and best-oxygenation strategy (93.3 ± 40.0 vs. 229.7 ± 159.8 and 187.4 ± 146.2 mm Hg, *p* < 0.01). Best-oxygenation PEEP provided highest Pao_2_/Fio_2_ (293 ± 137.8 mm Hg), whereas best-compliance PEEP provided higher Pao_2_/Fio_2_ than the ARDSnet low PEEP–Fio_2_ table (252 ± 130 vs. 204 ± 103 mm Hg). Paco_2_ was similar among the three strategies (*p* = 0.58). MAP was significantly higher with the PEEP guided by best compliance than MAP with the other two strategies (*p* < 0.001), whereas heart rate was similar (*p* = 0.96).

### Subgroup Analysis

We divided subjects into two subgroups (mild ARDS and moderate/severe ARDS) according to Pao_2_/Fio_2_ at study enrollment based on Berlin Definition (27). Characteristics of the subjects, including gender, age, SOFA score, and duration of mechanical ventilation were similar between two subgroups. Pao_2_ and Pao_2_/Fio_2_ were higher in the mild ARDS group than those in the moderate/severe ARDS group. *V*_T_, Fio_2_, Paco_2_, Pplat, PEEP, driving pressure, *C*_rs_, MAP, and heart rate at baseline (before initiation of the protocol) were not different between the two groups (Table 1).

**Figure 3:**
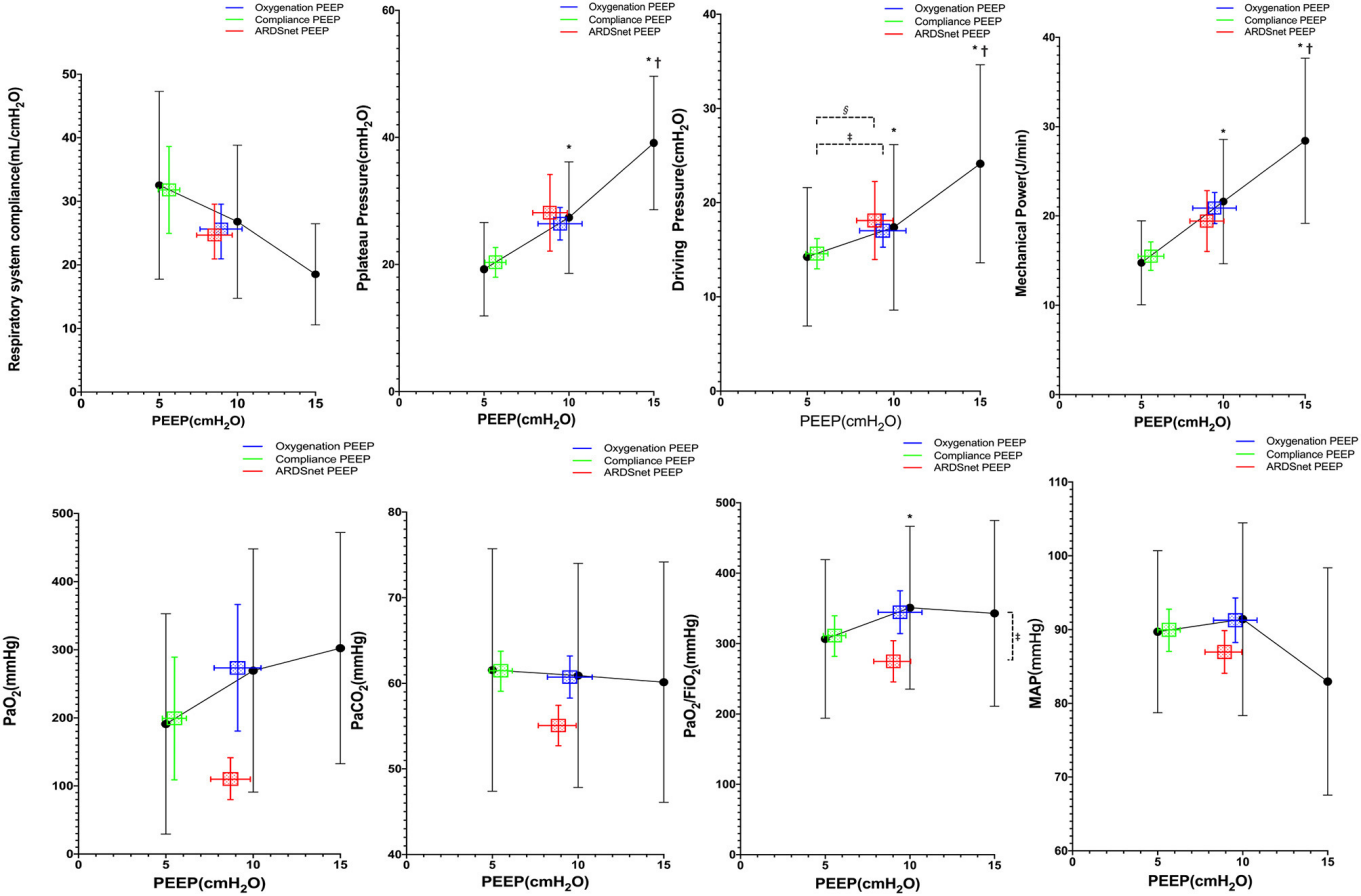
Reponses to PEEP in subjects with mild ARDS (*n* = 8). *vs. PEEP 5 cm H_2_O, *p* <0.05; ^†^vs. PEEP 10 cm H_2_O, *p* < 0.05; ^‡^vs. oxygenation PEEP, *p* < 0.05; ^§^vs. ARDSnet PEEP, *p* < 0.05.

There were eight subjects in the study with mild ARDS at study enrollment. Pplat, driving pressure, and mechanical power decreased significantly from PEEP 15 to 5 cm H_2_O (Figure 3). PEEP levels were not significantly different among the three PEEP strategies. Pao_2_, Pplat, *C*_rs_, and mechanical power were not different among the three PEEP strategies (Figure 3). However, driving pressure at best-oxygenation PEEP and that at ARDSnet PEEP were higher than driving pressure at best-compliance PEEP (16 ± 8 and 18 ± 11 vs. 14 ± 7 cm H_2_O, *p* = 0.03). There was one subject with missing ABG results. Paco_2_, MAP, and HR at PEEP guided by the three strategies were similar (Figure 3).

**Figure 4:**
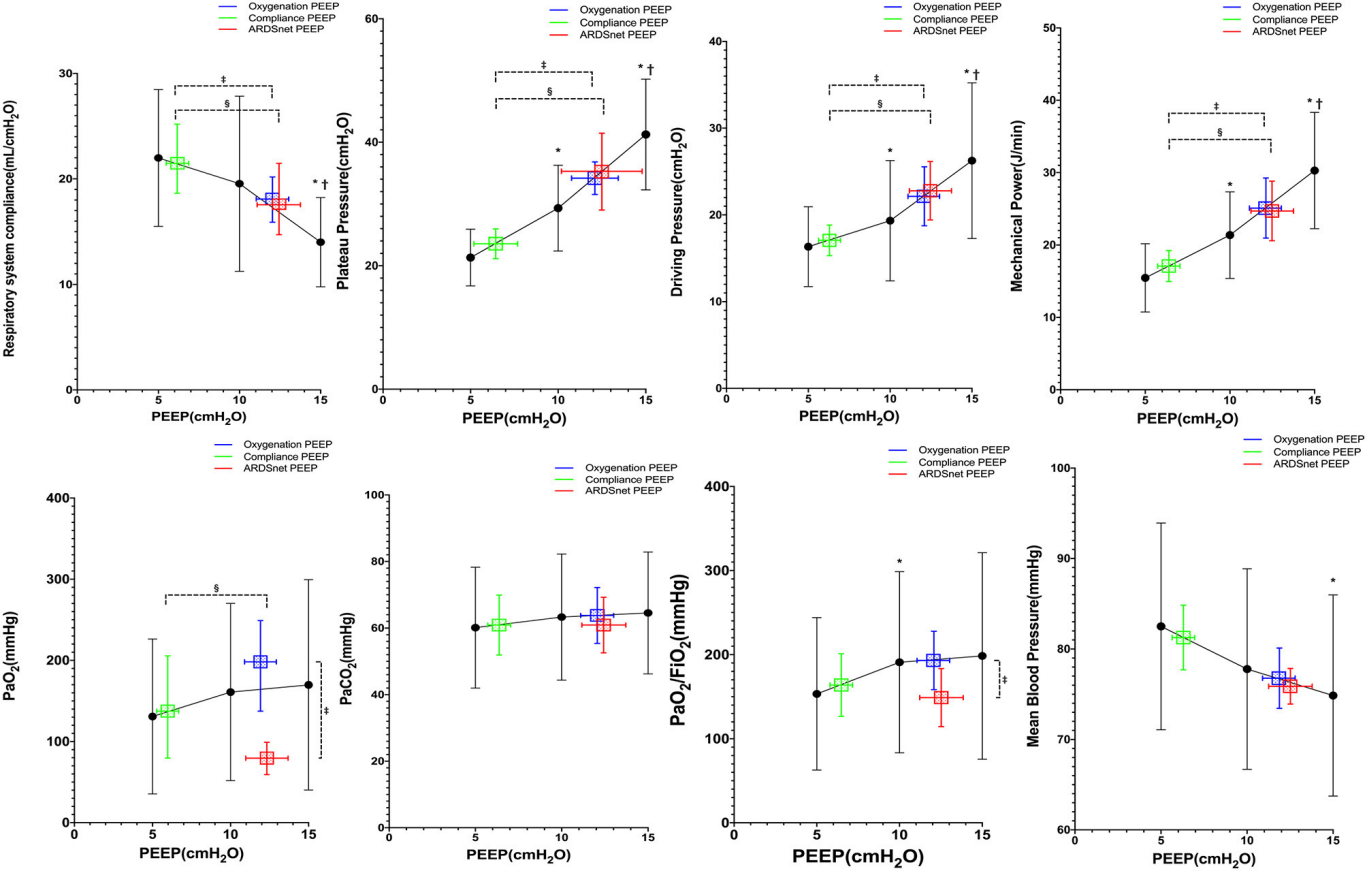
Reponses to PEEP in subjects with moderate or severe ARDS (*n* = 12). *vs. PEEP 5 cm H_2_O, *p* < 0.05; ^†^vs. PEEP 10 cm H_2_O, *p* < 0.05; ^‡^vs. oxygenation PEEP, *p* < 0.05; ^§^vs. ARDSnet PEEP, *p* < 0.05.

There were 12 subjects with moderate/severe ARDS at study enrollment. Pplat, driving pressure, and mechanical power decreased significantly from PEEP 15 to 5 cm H_2_O (Figure 4), whereas MAP increased significantly at lower PEEP (Figure 4). ARDSnet PEEP and best-oxygenation PEEP were higher than the best-compliance PEEP (12 ± 7 and 12 ± 4 vs. 6 ± 2 cm H_2_O, *p* = 0.007). Pplat, driving pressure, and mechanical power at best-oxygenation PEEP and ARDSnet PEEP were higher than at best-compliance PEEP (Pplat: 35 ± 12 and 36 ± 15 vs. 22 ± 14 cm H_2_O, *p* = 0.002; driving pressure: 23 ± 10 and 24 ± 10 vs. 16 ± 5 cm H_2_O, *p* = 0.002; mechanical power: 26 ± 11 and 25 ± 11 vs. 16 ± 5 J/min, *p* = 0.004) (Figure 4). *C*_rs_ at best-compliance PEEP was higher than those at best-oxygenation PEEP and ARDSnet PEEP (23.0 ± 7.8 vs. 16.6 ± 4.8 and 16.7 ± 7.5 ml/cm H_2_O, *p* < 0.01). There were three subjects without ABG measurements at PEEP 15 cm H_2_O as previously mentioned. Pao_2_ and Pao_2_/Fio_2_ were lower with ARDSnet PEEP. pH, Paco_2_, MAP, and HR between the three strategies were similar.

### Potential Risk of VILI

In mild ARDS subjects, the potential risks of lung injury induced by three PEEP strategies were similar and relatively low (Table 2). In moderate/severe ARDS subjects, however, both best-oxygenation PEEP and ARDSnet PEEP generated “injurious” Pplat (>30 cm H_2_O) and mechanical power (>25 J/min) more frequently than best-compliance PEEP (Table 2).

**Table 2:** Incidence of potentially high risk of ventilator-induced lung injury.

	**Mild ARDS (*****n*** **=** **8)**			**Moderate or severe ARDS (*****n*** **=** **12)**		
	**Best-oxygenation**	**Best-compliance**	**ARDSnet low PEEP table**	**Best-oxygenation**	**Best-compliance**	**ARDSnet low PEEP table**
P_plat_ > 30 cm H_2_O *n* (%)	1 (12.5%)	1 (12.5%)	2 (25.0%)	8 (66.7%)^*^	1 (8.3%)	8 (66.7%)^†^
ΔP > 15 cm H_2_O *n* (%)	3 (27.5%)	2 (25.0%)	3 (27.5%)	10 (83.3%)	5 (41.7%)	8 (66.7%)
Mechanical Power > 25 J/min *n* (%)	2 (25.0%)	0	1 (12.5%)	6 (50.0%)^*^	0	7 (58.3%)^†^

*Pplat, plateau pressure; ΔP, driving pressure*.

* *p < 0.05 by Fisher's exact tests in best-oxygenation PEEP vs. best-compliance PEEP*.

† *p < 0.05 by Fisher's exact tests in ARDSnet low PEEP table vs. best-compliance PEEP*.

## Discussion

The main findings of the present study are that in COVID-19–induced ARDS subjects mechanically ventilated in Wuhan, China, most of the subjects had a poor response to high PEEP. The PEEP/Fio_2_ table and best-oxygenation PEEP had similar effects to best-compliance PEEP on gas exchanges and hemodynamics, but there was an increased risk of lung injury due to high PEEP levels particularly for the subjects with moderate/severe ARDS.

Lung recruitability of COVID-19–induced ARDS is highly variable. Our previous study assessed the potential for lung recruitment through the recruitment-to-inflation ratio (28), showing that the majority of the cohort had poor recruitability to high PEEP in subjects with COVID-19–induced ARDS (19). In two recent studies by Beloncle et al. and Mauri et al., however, the potential for lung recruitment (assessed by the recruitment-to-inflation ratio) of subjects with COVID-19–induced ARDS in Italy and France was much higher than what we found in Wuhan, with larger intersubject variability (17, 18). Gattinoni and Marini proposed a hypothesis that COVID-19 pneumonia subjects should be divided into different phenotypes to offer different respiratory support (20–22, 29, 30). Subjects in the present study had poor response to high PEEP (dramatic increase in Pplat, driving pressure, mechanical power with reduction in MAP). These poor responses to high PEEP suggest poor lung recruitability in these subjects, which is consistent with our previous study in which lung recruitability was directly measured (19) and other studies (31–35). However, subjects in our study also presented with low baseline compliance, which were not the proposed “type H phenotype” patients and differ from other studies.

We suspect that the differences in lung recruitability and compliance among studies might be caused by different durations of invasive ventilation prior to study enrollment. The subjects in our study received relatively long durations of invasive mechanical ventilation before measurement (11 ± 6 days), which can generate progressive lung fibrosis and thus worsen the compliance and lower the lung recruitability. The time course of changes in mechanics has been well-illustrated by (29). Alternatively, these differences might be related to the different lineages of coronavirus (36–39).

Limiting the risk of hyperinflation of the “baby lung” when applying high PEEP to promote the recruitment of the collapsed lung is essential. The present study showed that the PEEP selected by ARDSnet low PEEP–Fio_2_ table or the best-oxygenation methods was significantly higher than the PEEP selected by the best-compliance method. As a consequence, both ARDSnet low PEEP–Fio_2_ table and the best-oxygenation strategies led to higher Pplat, driving pressure, and mechanical power. Although the thresholds of the limits of these parameters can be debatable, the present study showed that the incidences of “injurious” Pplat (>30 cm H_2_O) and mechanical power (>25 J/min) were higher in ARDSnet low PEEP–Fio_2_ table and the best-oxygenation strategy in subjects with moderate or severe ARDS. Although we did not test the ARDSnet high PEEP–Fio_2_ table in our study, it obviously would have increased the risk of overdistension in our subjects. By contrast, the best-compliance strategy did not bring notable risk of overdistension in mild or moderate/severe ARDS subjects.

There are some limitations in our study. (1) Although it is a multicenter study, the sample size is less than expected because of a dramatic reduction in the number of newly admitted ICU subjects during the study. (2) Duration of mechanical ventilation of subjects involved after intubation was 11 ± 6 days in this study. Caution is required for comparing our results with other studies, which enrolled subjects in an earlier phase of mechanical ventilation. (3) We did not assess lung recruitability directly. Instead, we assessed the change in respiratory mechanics, gas exchange, and hemodynamic effects during the PEEP trial, which reflect the subjects' response to PEEP. (4) We simplified the PEEP trial compared to other studies, which used a decremental PEEP trial by 2 cm H_2_O after a recruitment maneuver. (5) We did not measure biomarkers at different PEEP settings, which can help us better assess the risk of VILI.

In our cohort with COVID-19–induced ARDS from Wuhan, the ARDSnet low PEEP/Fio_2_ table and the best-oxygenation strategies provide higher PEEP than the best-compliance strategy. Our subjects had poor responses to high PEEP as high PEEP often led to excessive Pplat, driving pressure mechanical power, and worse MAP. Further studies on the effects of PEEP on COVID-19–induced ARDS are needed to confirm our findings.

## Data Availability Statement

The original contributions generated for the study are included in the article/supplementary material, further inquiries can be directed to the corresponding author/s.

## Ethics Statement

The studies involving human participants were reviewed and approved by Wuhan Jinyintan Research Ethics Board. Written informed consent for participation was not required for this study in accordance with the national legislation and the institutional requirements.

## Author Contributions

BD and HQ: conceptualization, methodology, project administration, visualization, funding acquisition, and supervision. CP and CL: formal analysis. CP, XS, HR, HW, LX, YH, and JX: investigation. CP, CL, and LC: writing—original draft. CP, CL, YY, and LC: writing—review and editing. All authors have read and agreed to the published version of the manuscript.

## Conflict of Interest

The authors declare that the research was conducted in the absence of any commercial or financial relationships that could be construed as a potential conflict of interest.
